# Estimating a neutral reference for electroencephalographic recordings: the importance of using a high-density montage and a realistic head model

**DOI:** 10.1088/1741-2560/12/5/056012

**Published:** 2015-08-26

**Authors:** Quanying Liu, Joshua H Balsters, Marc Baechinger, Onno van der Groen, Nicole Wenderoth, Dante Mantini

**Affiliations:** 1 Neural Control of Movement Laboratory, ETH Zurich, 8057 Zurich, Switzerland; 2 Department of Experimental Psychology, University of Oxford, Oxford OX1 3UD, UK; 3 Trinity College Institute of Neuroscience and School of Psychology, Trinity College, Dublin 2, Ireland; 4 Laboratory of Movement Control and Neuroplasticity, KU Leuven, B-3001 Leuven, Belgium; dante.mantini@hest.ethz.ch

**Keywords:** electroencephalography, head model, average reference, reference electrode standardization, montage, neural activity, electrode position

## Abstract

*Objective*. In electroencephalography (EEG) measurements, the signal of each recording electrode is contrasted with a reference electrode or a combination of electrodes. The estimation of a neutral reference is a long-standing issue in EEG data analysis, which has motivated the proposal of different re-referencing methods, among which linked-mastoid re-referencing (LMR), average re-referencing (AR) and reference electrode standardization technique (REST). In this study we quantitatively assessed the extent to which the use of a high-density montage and a realistic head model can impact on the optimal estimation of a neutral reference for EEG recordings. *Approach*. Using simulated recordings generated by projecting specific source activity over the sensors, we assessed to what extent AR, REST and LMR may distort the scalp topography. We examined the impact electrode coverage has on AR and REST, and how accurate the REST reconstruction is for realistic and less realistic (three-layer and single-layer spherical) head models, and with possible uncertainty in the electrode positions. We assessed LMR, AR and REST also in the presence of typical EEG artifacts that are mixed in the recordings. Finally, we applied them to real EEG data collected in a target detection experiment to corroborate our findings on simulated data. *Main results*. Both AR and REST have relatively low reconstruction errors compared to LMR, and that REST is less sensitive than AR and LMR to artifacts mixed in the EEG data. For both AR and REST, high electrode density yields low re-referencing reconstruction errors. A realistic head model is critical for REST, leading to a more accurate estimate of a neutral reference compared to spherical head models. With a low-density montage, REST shows a more reliable reconstruction than AR either with a realistic or a three-layer spherical head model. Conversely, with a high-density montage AR yields better results unless precise information on electrode positions is available. *Significance*. Our study is the first to quantitatively assess the performance of EEG re-referencing techniques in relation to the use of a high-density montage and a realistic head model. We hope our study will help researchers in the choice of the most effective re-referencing approach for their EEG studies.

## Introduction

Electroencephalography (EEG) is a non-invasive approach of measuring brain activity at high temporal resolution using electrodes placed on the subject’s scalp. This technique has been widely used to investigate such domains as perception, cognition, emotion and attention, given that it can provide great insights into the neural temporal dynamics underlying these processes (Carballogonzalez *et al* 1989, van der Lubbe *et al* 2000, Silberstein *et al* 2001, Porcaro *et al* 2009). An important aspect that needs to be considered when evaluating EEG results is that EEG is a relative measure that compares the recording site with another (reference) site. The most commonly used physical references are the vertex (Cz) reference (Mulert *et al* 2008), the (left or right) mastoid reference and the earlobe reference (Flanigan *et al* 1995). All of these were suggested to be far enough from all brain sources to be considered zero-potential references. However, there is no such point on human body surface (Yao 2001, Nunez and Srinivasan 2006). All physical electrode sites involve physiological dynamic processes that will inevitably affect the temporal dynamic analysis and spectral analysis of the EEG signal because a non-constant temporal component is added to it (Yao 2001, Qin *et al* 2010, Thatcher 2012). The voltage waveforms and power distribution are not unique because of their dependence on the choice of a reference, which may result in quite different conclusions from the same experiment depending on the reference used (Joyce and Rossion 2005, Tian and Yao 2013). Therefore, one critical issue for EEG is to eliminate—or at least minimize—the effects of residual neuronal activity in the physical reference by re-referencing the data (Kayser and Tenke 2010). This can be done by estimating the signal in a virtual reference, typically combining information from multiple electrodes, and subtracting it from all recordings.

Several re-referencing schemes were introduced in previous studies. A popular one is the linked-mastoid re-referencing (LMR), which is obtained by linearly combination of the potentials measured at mastoid sites (Kornhuber and Deecke 1965, Nunez *et al* 1997). However, this reference strategy has been criticized given that it leads to a substantial shift of high power regions towards frontal and central positions (Yao *et al* 2005). This spatial distortion problem may strongly limit the neurophysiological interpretability of the EEG results. Another—potentially less biased—re-referencing approach is the use of the average re-referencing (AR). This defines the virtual reference as the average of all EEG signals (Offner 1950). The underlying principle of AR is that EEG potentials nearly sum to zero if the head is modelled as a closed surface, with the electrodes being densely sampled over it (Bertrand *et al* 1985, Nunez *et al* 1997). However, the limited electrode density and incomplete electrode coverage (the bottom half of the head is not completely sampled) result in the neutral point being estimated as being higher up on the head than it should be for vertically oriented fields (Desmedt and Tomberg 1990). There is no doubt that the EEG montage influences AR, but it is still unclear to what extent this is the case. The reference electrode standardization technique (REST) was proposed as being able to overcome the limitations of AR (Yao 2001, Yao *et al* 2005). The general idea behind REST is that, since EEG source localizations do not depend on the chosen reference, it is possible to rely on them to reconstruct the reference as being at infinite distance. Although REST may be in principle more accurate than alternative approaches, a potential limitation is the sensitivity to the head model used, which is strictly required for source localizations. Furthermore, REST, as well as AR, is dependent on both electrode density and coverage. Even though REST has been shown in several studies to be potentially more accurate than AR in many situations (Marzetti *et al* 2007, Qin *et al* 2010, Khodayari-Rostamabad *et al* 2013, Xu *et al* 2014), it is unclear if this can still be the case when no high-density montage is used or no precise information about the structure of the subject’s head (Valdes-Hernandez *et al* 2009) and the relative position of the EEG electrodes (Russell *et al* 2005) is available. Accordingly, the choice of a most appropriate EEG re-referencing technique remains an open question.

In this study we evaluated the importance of using a high-density EEG system, and collecting accurate information about electrode positions and the head structural image, to conduct event related potential (ERP) analyses. These aspects are particularly relevant for REST, but can partially affect the AR technique as well. LMR, which is independent of the EEG montage and the head model, was included in the study for comparison. Using simulated recordings generated by projecting specific source activity over the sensors, we assessed to what extent the AR, REST and LMR may distort the scalp topography. We examined what impacts the electrode coverage has on AR and REST, and how accurate the REST reconstruction is for realistic and less realistic (spherical) head models, and with possible uncertainty in the electrode positions. In addition, we used simulated data to evaluate the accuracy of AR, LMR and REST in the presence of typical EEG artifacts that are mixed in the recordings. Finally, we applied REST and AR to real EEG data collected in a target detection experiment to corroborate our findings on simulated data.

## Methods

### Simulation

**Figure 1.**
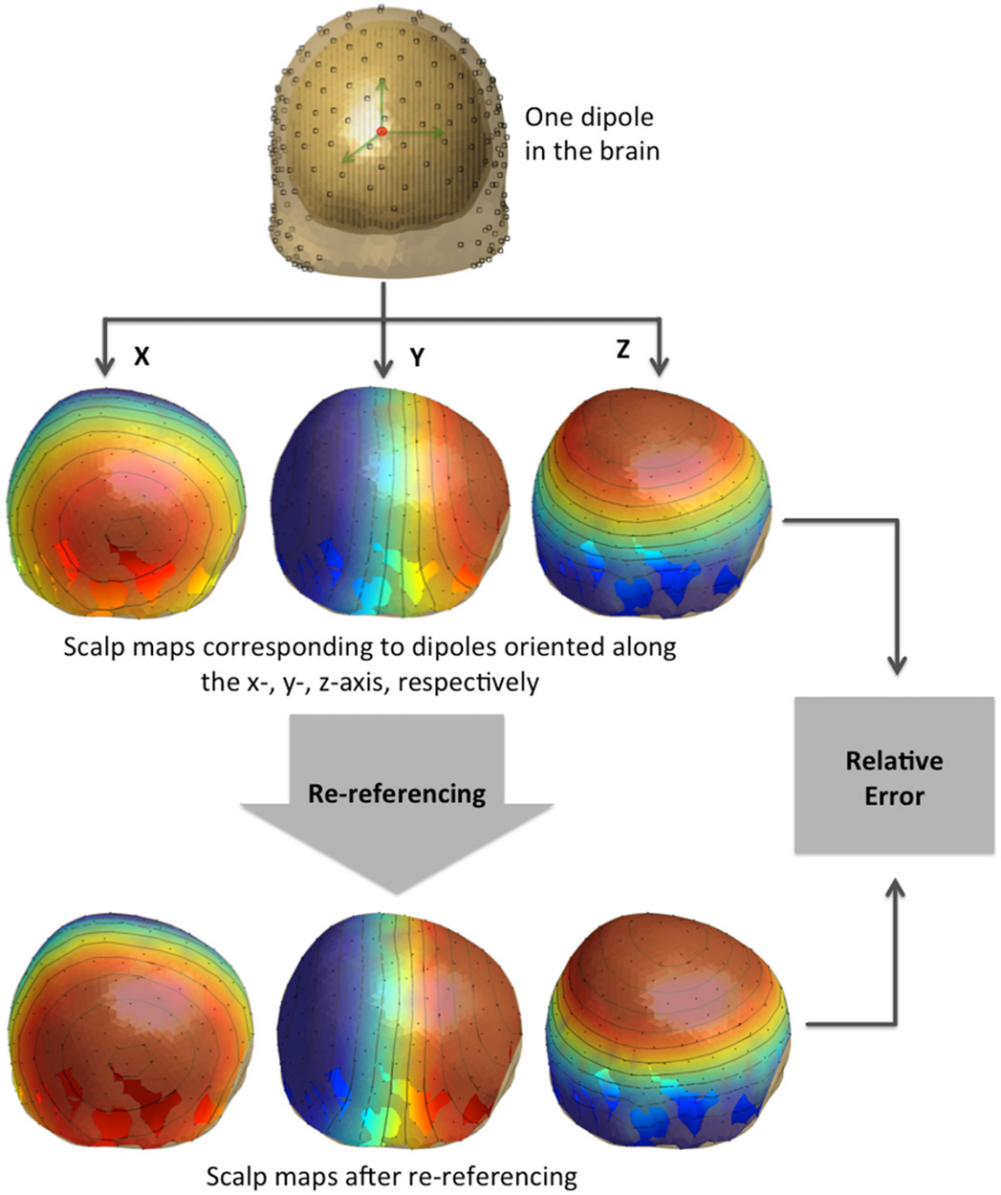
Workflow for the generation and analysis of simulated EEG data: the head model is built based on the structural MR image, and then the simulated scalp potential is calculated from the one dipole with orientations along the *x*-, *y*- or *z*-axis respectively. The re-referencing technique is then applied to the simulated scalp signals. The resulting intensities are compared with the original ones based on the relative error.

The workflow of our simulation, which we used to assess the characteristics of different re-referencing approaches, is illustrated in figure 1. In short, we built a realistic head model by using a structural magnetic resonance image (Holmes *et al* 1998). We registered EEG sensors to the scalp, and we modelled EEG sources as dipoles arranged as a uniform 3D grid covering the whole brain. We generated EEG scalp maps by calculating the potentials over the sensors associated with single dipolar sources. Each dipolar source was oriented along the *x*-, *y*- or *z*-axis, respectively. We then applied a re-referencing technique to the scalp map, and assessed the correspondence between the original potentials and those after re-referencing. In this framework, the best re-referencing technique is the one that minimally alters the original scalp map, i.e. the one that yields the smallest relative error (RE).

#### Generation of simulated EEG potentials

First, we created a template head model in volumetric space by using a three-layer boundary element method (BEM). To define the head compartments (i.e. brain, skull, skin), we used the Colin27 image, which is the magnetic resonance image (Holmes *et al* 1998) included in SPM8 (Wellcome Trust Centre for Neuroimaging, London, UK). The conductivities of scalp, skull and brain were set to 1, 0.0125 and 1 s m^−1^, respectively (Rush and Driscoll 1968, Ahlfors *et al* 2010b). Electrode positions for a 256-channel Electrical Geodesics (Eugene, USA) system, which were defined in the same space, were also extracted from SPM8. Using information from the head model and the electrode positions, we generated a leadfield matrix *G* using the BEMCP approach (Phillips 2000). This matrix has dimension [3*M* × *N*], where *M* is the number of brain voxels and *N* is the number of channels, and permits to reconstruct the sources *S* along the *x*-, *y*- and *z*-directions from the recordings. The relationship between the sources *S* and the recordings *V* can be modelled by using the equation:

1 \begin{eqnarray*}V=G\cdot S.\end{eqnarray*}

Notably, the scalp map associated with a specific dipole position and orientation can be obtained by selecting the corresponding row in the leadfield matrix (Yao 2001). Due to the linear relationship between *V* and *S* expressed in equation (1), we considered only one time sample in our analysis as representative for the whole EEG (and ERP) time courses. To assess the performance of the re-referencing techniques unbiased by the position of the dipole, we analysed 100 dipole positions randomly selected across the brain space.

#### Re-referencing techniques

The re-referencing technique is based on the idea of a reference

2 \begin{eqnarray*}{V}_{{\rm{re}}-{\rm{ref}}}=V-{V}_{{\rm{ref}}}={R}_{{\rm{re}}-{\rm{ref}}}\cdot V,\end{eqnarray*}

where *V* is the measured scalp signal, *V*_ref_ is the reference signal, *V*_re–ref_ is the re-referenced signal and *R*_re*–*ref_ is the re-referencing (or transfer) matrix.

As for LMR, the reference signal ${V}_{{\rm{ref}}}=\frac{1}{2}({v}_{{\rm{LM}}}+{v}_{{\rm{RM}}}),$ where *v*_LM_ and *v*_RM_ are the signals for the left and right mastoid channels respectively. The re-referencing matrix to be used in the formula ${V}_{{\rm{LMR}}}={R}_{{\rm{LMR}}}\cdot {V}_{M}$ can be written as ${R}_{{\rm{LMR}}}=I-{K}_{{\rm{LMR}}}.$ *I* is an identity matrix with *N*-by-*N* dimension (*N* is the number of channels), and *K*_LMR_ is a *N*-by-*N* matrix with all elements equal to 0 except for the columns of the left and right mastoid channels, which are equal to 0.5.

As for AR, the reference signal is ${V}_{{\rm{ref}}}=\frac{1}{2}\sum _{i=1}^{N}{v}_{i},$ where *v*_*i*_ is the *i*th channel of brain signal. The re-referencing matrix to be used in the formula ${V}_{{\rm{AR}}}={R}_{{\rm{AR}}}\cdot V$ can be written as ${R}_{{\rm{AR}}}=I-{K}_{{\rm{AR}}},$ with *K*_AR_ being a *N*-by-*N* matrix with all elements equal to 1/*N*.

REST is a mathematical method that approximately transforms the EEG recordings with a scalp point reference to recordings with a reference at infinity. The latter is meant to be a point far from all the possible neural electric sources (Yao 2001, Yao *et al* 2007). The reconstructed signal referenced at infinity, namely *V*_REST_, can be modelled as:

3 \begin{eqnarray*}{V}_{{\rm{REST}}}=G\cdot \hat{S},\end{eqnarray*}

where $\hat{S}$ is the estimate of source *S* in equation (1), and *G* is the lead-field matrix. Based on the equivalent source technique (EST), the inverse problem solution is not affected by the choice of the reference (Pascual-Marqui and Lehmann 1993, Geselowitz 1998). Accordingly

4 \begin{eqnarray*}\hat{S}={G}^{+}\cdot V={G}_{{\rm{AR}}}^{+}\cdot {V}_{{\rm{AR}}},\end{eqnarray*}

where ${G}_{{\rm{AR}}}^{+}$ denotes the generalized Moore–Penrose inverse of demeaned *G* (Yao 2001, Yao *et al* 2007). Combining equation (3) and (4), we obtain

5 \begin{eqnarray*}{V}_{{\rm{REST}}}=G\cdot {G}_{{\rm{AR}}}^{+}\cdot {V}_{{\rm{AR}}}=G\cdot {G}_{{\rm{AR}}}^{+}\cdot {R}_{{\rm{AR}}}\cdot V.\end{eqnarray*}

Accordingly, the re-referencing matrix to be used in the formula ${V}_{{\rm{REST}}}={R}_{{\rm{REST}}}\cdot V$ can be calculated with the formula ${R}_{{\rm{REST}}}=G\cdot {G}_{{\rm{AR}}}^{+}\cdot {R}_{{\rm{AR}}}.$ Note that this matrix depends on the chosen head model, the location and the direction of the dipoles comprising the source model and the electrode coverage.

#### Performance measurement

The simulated EEG scalp map was compared with the one obtained after re-reference in terms of RE (Yao 2001, Marzetti *et al* 2007). In our study, we used two related but different measures: the global RE (gRE) for all channels together and the channel-based RE (cRE) for single-channel signals. The definition of gRE for a single time sample is as follows

6 \begin{eqnarray*}{\rm{gRE}}=\displaystyle \frac{\sqrt{\displaystyle {\sum }_{i=1}^{N}{({V}_{{\rm{re}}-{\rm{ref}}}(i)-V(i))}^{2}}}{\sqrt{\displaystyle {\sum }_{i=1}^{N}V{(i)}^{2}}}.\end{eqnarray*}

It should be noted that the value of gRE is always positive. In order to explore the RE distribution over the scalp and the increase or decrease of intensity for single-channel signals, we defined the cRE for the *i*th channel as follows:

7 \begin{eqnarray*}{\rm{cRE}}=\displaystyle \frac{{V}_{{\rm{re}}-{\rm{ref}}}(i)-V(i)}{V(i)}.\end{eqnarray*}

To examine the effectiveness of the re-referencing approaches for the different dipole locations and orientations, simulations were conducted for each voxel of a discrete cubic grid as a source position with each of the three unit dipoles (*Px*, *Py*, *Pz*) directed along the three Cartesian coordinate (*x*, *y*, *z*) directions separately. Since the transformations shown in equation (2) are linear, we only needed to check the performance according to the potential of a single dipole and noise independently.

#### Methods validation

First, we examined the amplitude distribution of the simulated EEG data before and after re-referencing. The referencing strategies used included LMR, AR and REST, which are the commonly used re-referencing methods in the EEG literature. The Cz reference (CzR) was included in the analysis as well. To assess the performance of the re-referencing techniques unbiased by the position of the dipole, we analysed 100 dipole positions randomly selected across the brain space. After examining the general features of the EEG signals using the four kinds of reference, we investigated the performance of AR and REST with respect to more specific aspects. First, we evaluated the impact of the number of channels available for the calculation of the new reference. To this end, we produced three additional montages with respect to the 256-channel one: a 21-channel montage corresponding to the 10/20 system, an 71-channel montage corresponding to the 10/10 system, and a 128-channel montage (see figure S1).

We subsequently analysed to what extent the head model influenced referencing performance. This analysis was only conducted on the REST data, given that the average reference is not affected by this factor. To conduct this analysis, we generated a single-sphere model and a three-concentric-sphere model (Rush and Driscoll 1968) in addition to the three-layer realistic BEM head model. The relative dimensions of the three concentric spheres were 0.87, 0.92 and 1, respectively. The conductivities were 1, 0.0125 and 1 s m^−1^, respectively. For each head model, we calculated the reference standardization matrix *R*_REST_ and then applied REST.

REST can be dependent on the accuracy of the EEG electrode positions. We also investigated this aspect by introducing either random or systematic across-electrode shifts of 1, 2 and 4 and 8 mm in the montage coordinates, and then applying REST. The systematic shifts were optionally corrected by rigid-body transformation using the headshape information. This was done using a dedicated algorithm implemented in SPM8. We generated ten different cases of each type of electrode shifts. For each realization, we measured the increase in the gRE introduced by these small errors in the electrode positions and then we averaged them to increase the accuracy of our estimates.

**Figure 2.**
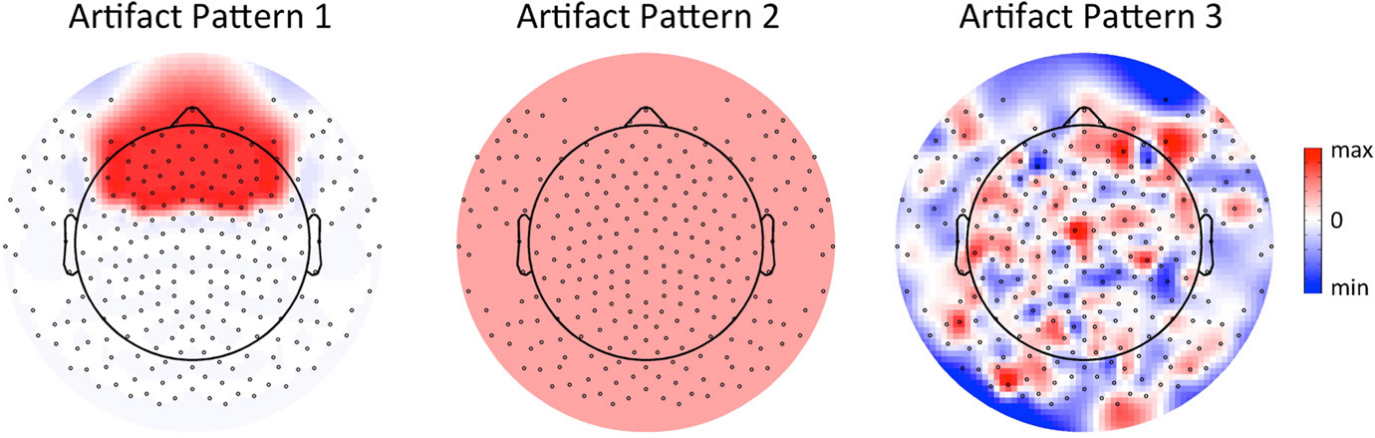
Topography of three kinds of artifactual patterns used in the simulations: localized over part of the scalp (artifact pattern 1), homogeneously distributed over the scalp (artifact pattern 2) and randomly distributed over the whole scalp (artifact pattern 3).

Finally, we tested the effect of non-neuronal signals mixed in the scalp EEG on the re-referencing results. Specifically, we investigated whether the re-referencing techniques under investigation, i.e. LMR, AR and REST, are influenced by artifacts, and to what extent they are capable of attenuating them. To address this question, we modified the EEG scalp maps generated by single dipoles, adding the following kinds of spatial patterns (figure 2): (1) a patch of activity concentrated over the frontal region, which is comparable with the non-neuronal activity generated the eye movements; (2) a homogeneously distributed signal over the scalp, which could mimic the spatial distribution of the 50 Hz power line; (3) uncorrelated Gaussian white noise all over the scalp. We calculated an artifact reduction index (ARI), which was defined as the ratio between the average artifact power across all channels, after and before re-referencing.

### EEG experiment

We also analysed actual ERP/EEG data to evaluate the quality of the signal reconstructed using the different re-referencing techniques. In particular, we were interested in understanding to what extent the signal intensity is preserved in the ERPs, and whether the noise level is also influenced.

#### Participants and task design

Seventeen right-handed subjects (age 27.6 ± 5.9 yr, 4 males and 13 females) participated in the experiment. All participants reported normal or corrected-to-normal vision, had no psychiatric or neurological history, were free of psychotropic or vasoactive medication. Before undergoing the examination, they gave their written informed consent to the experimental procedures, which were approved by the local Institutional Ethics Committee of ETH Zurich.

The EEG experiment was performed, in accordance with the approved guidelines, in a quiet, air-conditioned laboratory with soft natural light. The task consisted of a visual oddball paradigm, with the presentation of 80% of frequent stimuli and 20% of rare stimuli respectively (Mantini *et al* 2009). The subjects were asked to press the button as soon as possible after the rare event appeared (target). The stimuli consisted of purple circles of 3.2° and 1.6° of the visual angle for frequent and rare events respectively, appearing on a black background with 7 ms duration. The inter-stimulus interval was 2 s and with 3–5 frequent stimuli randomly presented between rare stimuli (6–10 s interval between rare targets) (O’Connell *et al* 2012, Murphy *et al* 2014). To reduce eye movements and blinks, subjects were instructed to keep fixation on the centre of screen during the experiment.

#### EEG data acquisition

EEG signals were recorded by the 256-channel HydroCel Geodesic Sensor Net using Ag/AgCl electrodes provided by Electrical Geodesics (EGI, Eugene, Oregon, USA). The EGI system uses the vertex (Cz) electrode as physical reference. EEG recordings were sampled online at 1000 Hz. All sensors and three fiducial positions were localized prior to the EEG acquisition by using a Geodesic Photogrammetry System (Russell *et al* 2005). Electric reference signals were collected in parallel to EEG data: those included electrooculograms for horizontal and vertical eye movements respectively, and an electromyogram for the muscular noise associated with swallowing.

The T1-weighted MRI of each subject was acquired in a separate experimental session using a Philips 3T Ingenia scanner with a turbo field echo sequence (Barttfeld *et al* 2014). The scanning parameters were: TR = 8.25 ms, TE = 3.8 ms, 8° flip angle, 240 × 240 × 160 field of view, 1 mm isotropic resolution.

#### EEG data analysis

A realistic forward model was created for each subject using a three-layer BEM applied to individual MRI data. Conductivities of brain, skull and skin were 1, 0.0125 and 1 s m^−1^. EEG electrodes were aligned on the head model using the locations of the three fiducials and the head shape extracted from the MR image, using the dedicated algorithm implemented in SPM8.

The experimental EEG data were filtered in the band 0.5–20 Hz and further processed using independent component analysis (ICA) for the removal of ocular and muscular artifacts (Mantini *et al* 2008). After ICA decomposition, the artifactual ICs were automatically detected by correlating their power time-courses with the power time courses of the electric reference signals: the horizontal electrooculogram, the vertical electrooculogram and the electromyogram (MEG) at the base of the neck. Then, we applied each of the different re-referencing techniques on the cleaned EEG data. Finally, we calculated ERPs for the rare events only, as these are the ones supposedly showing the P300 response (Picton 1992, Mantini *et al* 2009). Based on the results of the ERP analysis, we excluded one subject for which the P300 response was not clearly visible.

Using the preprocessed EEG signals, we measured the P300 activity and the noise levels on the averaged data. The P300 activity was defined as the maximum amplitude in the post-stimulus (from 200 to 500 ms) interval, while the noise amplitude was calculated as the average root mean square of the signal corresponding to the pre-stimulus (from −200 to 0 ms) interval. We conducted this analysis for each single channel, but we then focused on 41 electrodes over the parietal region (see figure S2), where the P300 (P3b) activity is typically most prominent (Picton 1992).

## Results

### Simulated data

**Figure 3.**
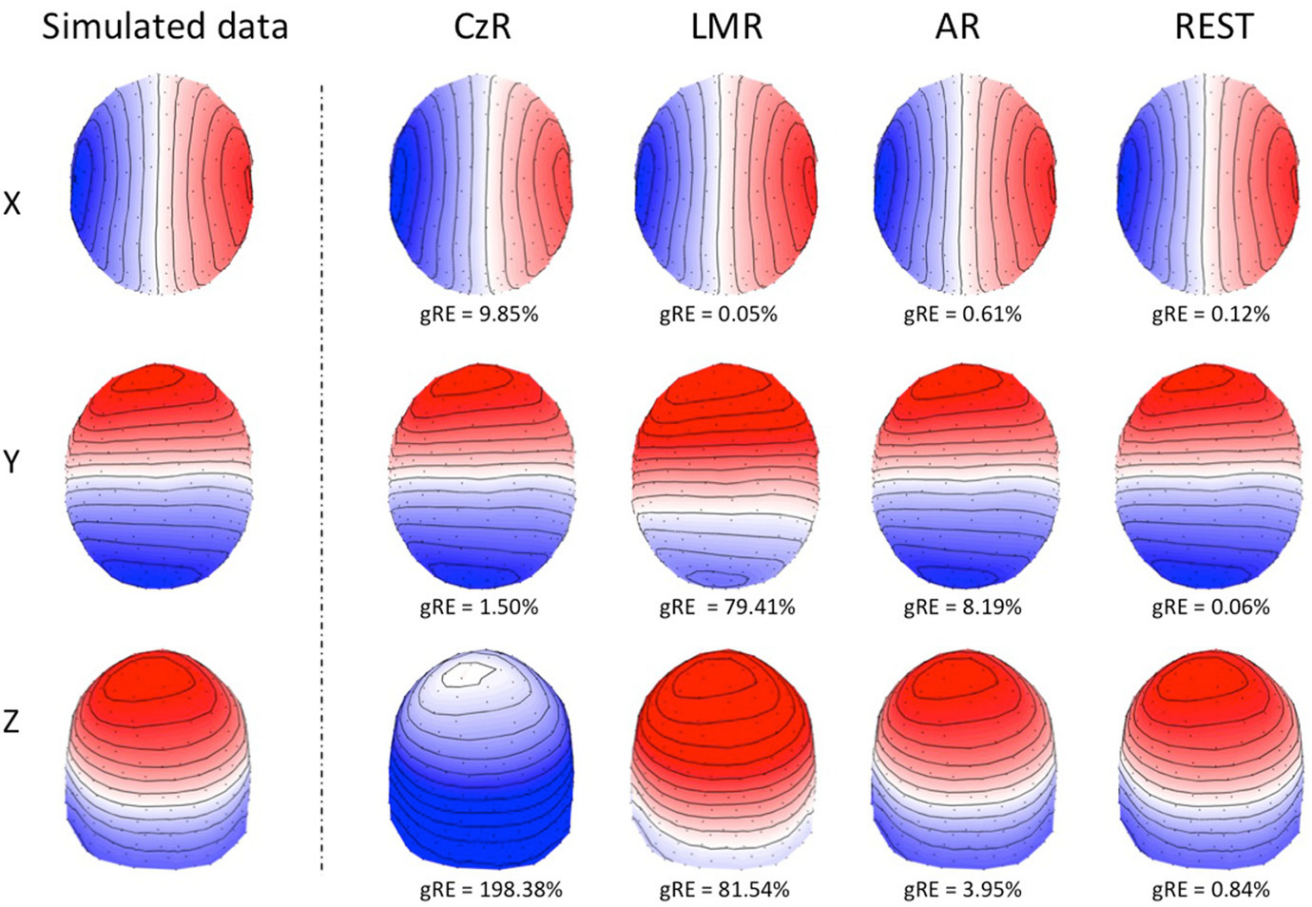
Effect of EEG data referencing. This illustrative example shows the scalp map generated by a dipolar source in the thalamus (MNI coordinates [−6 −13 5]), with orientation along the *x, y*, *z*-axis respectively. The simulated data, which are reference-free, are compared with those obtained using the CzR, LMR, AR and REST.

First, we examined the properties of different EEG re-referencing methods, specifically LMR, AR and REST, by means of simulated data. We generated a set of unbiased scalp maps by calculating the sensor potentials corresponding to single dipoles in the brain, and then we compared them to the same after re-referencing. This allowed us to quantify the error induced by the specific re-referencing method, both globally (gRE) and locally (cRE). Importantly, we assessed the characteristics of different re-referencing approaches considering the cases where the dipole is oriented along the *x*-, *y*- or *z*-axis (figure 1). By using a common colorbar for all references, we revealed that AR and REST provide scalp maps that are qualitatively similar to the original map, whereas significant distortions characterize both CzR and LMR. In particular, much less accurate reconstructions were obtained for dipoles oriented along the *y*-axis than the *x*-axis, and even less for those along the *z*-axis (figures 3 and S3). When we analysed the results of LMR, AR and REST in a quantitative manner, we also noticed that much lower errors could be achieved with REST compared to the other methods. This result did not depend on the orientation of the dipolar source (table 1).

**Table 1.** Global relative error (gRE) values for CzR, LMR, AR and REST reconstructions. The values in the table are the average gRE and its standard error (in brackets) across 100 dipoles at different locations of the brain, and oriented along the *x*-, *y*-, *z*-axis respectively.

	*X*	*Y*	*Z*	*XYZ*
CzR	35.65% (2.44%)	40.91% (3.12%)	156.84% (5.81%)	77.82% (2.55%)
LMR	31.67% (5.22%)	57.21% (3.84%)	76.25% (3.31%)	55.04% (2.64%)
AR	3.22% (0.30%)	7.13% (0.32%)	4.96% (0.39%)	5.10% (0.22%)
REST	0.64% (0.12%)	0.45% (0.08%)	0.66% (0.09%)	0.58% (0.06%)

We also analysed the spatial distribution of the potential alterations induced by the different re-referencing method. In doing so, we focused on AR and REST, which are the two approaches with relatively better performance. Our analysis confirmed that, in general, AR altered signal amplitudes more than REST. More importantly, it revealed that AR generally led to signal increases over the frontal regions and decreases over central, parietal and occipital regions (table 2). The increases/decreases depended not only on the locations of the active sources but also on their orientation (for examples, see figures S4 and S5).

**Table 2.** Channel-based relative error (cRE) for different brain regions, calculated for AR and REST respectively. The values shown in the table are the average cRE and standard error (in brackets) across 100 dipoles at different locations of the brain, and oriented along the *x*-, *y*-, *z*-axis respectively. Negative values, indicating a reduction of signals after re-referencing compared to the simulated data, are shown in bold.

		*X*	*Y*	*Z*	*XYZ*
Frontal	AR	2.32% (0.75%)	7.14% (0.63%)	−5.53% (1.75%)	1.31% (0.73%)
	REST	0.48% (0.21%)	0.10% (0.08%)	−0.32% (0.21%)	0.09% (0.08%)
Central and parietal	AR	1.98% (0.56%)	−2.15% (1.26%)	−2.09% (0.71%)	−0.75% (0.53%)
	REST	0.23% (0.18%)	0.033% (0.12%)	−0.16% (0.10%)	0.03% (0.10%)
Occipital	AR	−2.92% (4.20%)	−5.50% (0.41%)	−5.13% (1.92%)	−4.52% (1.54%)
	REST	−0.18% (0.45%)	0.01% (0.07%)	0.49% (0.49%)	0.11% (0.22%)

**Figure 4.**
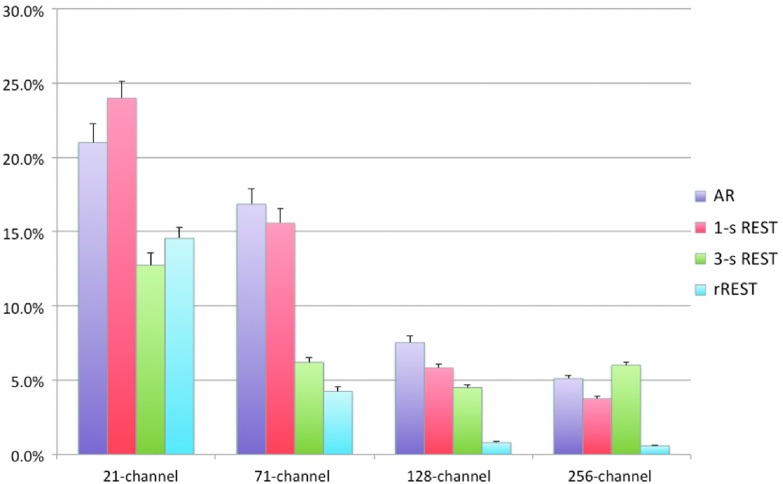
Accuracy of AR and REST with different EEG montages and head models. The bar plots show the average gRE and its standard error of re-referenced EEG data across 100 dipoles, obtained using 21-channel, 71-channel, 128-channel, 256-channel montages, respectively. The average gRE of AR and REST from one-layer spherical model (1-s), three-layer spherical (3-s) model and realistic (r) three-layer BEM are calculated for each of the four montages.

Then, we investigated to what extent the electrode density and coverage affect the AR and REST performances, as well as to what extent the accuracy of the head model affects the REST performance (figure 4). As expected, the AR and REST reconstructions were more precise with an increasing number of EEG channels. REST provided better results with a realistic head model than with one-layer and three-layer spherical models, except in the case of a 21-channel montage. The reconstruction error of REST with a realistic head model was always lower than that of AR. When a three-layer spherical model was used, the performance of REST was better than that of AR for the 21-channel, 71-channel and 128-channel systems, but not for the 256-channel system.

**Figure 5.**
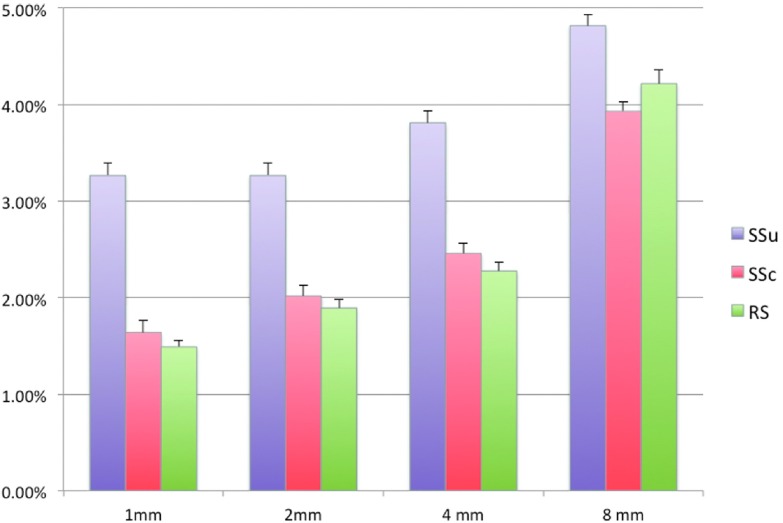
Accuracy for REST in the presence of noise in the electrode positions. The graph shows descriptive statistics about the increase of global relative error obtained by systematic shifts either corrected (SSc) or not (SSu) and random shifts (RS) across 100 dipoles oriented along the *x*-, *y*- and *z*-axis, with noise level equal to 1, 2, 4 and 8 mm. This analysis was conducted for 10 different realizations, using a three-layer BEM and an EEG montage with 256 channels.

The former results were obtained in ideal conditions, without considering the effects of noise. On the other hand, different sources of noise can affect the performances of AR and REST and account, at least in part, for the differences between them. Notably, in contrast to AR, the results of REST depended on the accuracy of electrode position information. We assessed this aspect by measuring the reconstruction error obtained after adding either random or systematic shifts to the electrode positions. We also examined to what extent error induced by systematic shifts could be attenuated by using the headshape information, as extracted from the structural image of the subject’s head. As expected, the level of noise negatively impacted on the reconstruction performances (figures 5 and S6). This effect was more pronounced for the 256-channel montage than for the 21-channel and 71-channel montages. Furthermore, REs induced by systematic shift without headshape correction were the most prominent, and reduced to levels comparable to those of random shifts when headshape correction was applied. Also notably, for the 256-channel montage the RE of REST obtained with electrode position uncertainty above 2 mm was comparable to the RE that characterized AR (i.e. 5.1%).

**Figure 6.**
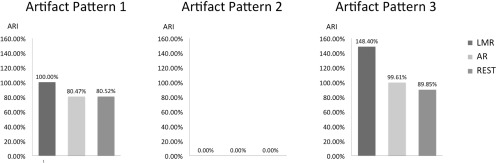
Artifact reduction index (ARI) for three kinds of artifactual patterns: localized over part of the scalp (artifact pattern 1), homogeneously distributed over the scalp (artifact pattern 2) and randomly distributed over the whole scalp (artifact pattern 3). The bar plots show the corresponding ARI for LMR, AR and REST, respectively.

Furthermore, we investigated the effects of non-neuronal signals added to the EEG data, thereby testing whether the re-referencing techniques are able to attenuate noise and artifacts and whether their performances depend on data quality. We analysed three different artifact spatial patterns: (1) localized over part of the scalp (patch); (2) homogeneously distributed over the scalp; (3) randomly distributed over the whole scalp (figure 2). This analysis revealed that the patched pattern could not be eliminated by LMR, but its power could be attenuated by about 20% both by AR and REST (figure 6). Furthermore, when dealing with a randomly distributed spatial artifact, REST led to a limited reduction of the artifact power, AR left the signals largely unchanged and LMR even yielded a substantial artifact power increase. Finally, the homogenous artifact pattern could be completely eliminated by LMR, AR and REST.

### Experimental data

**Figure 7.**
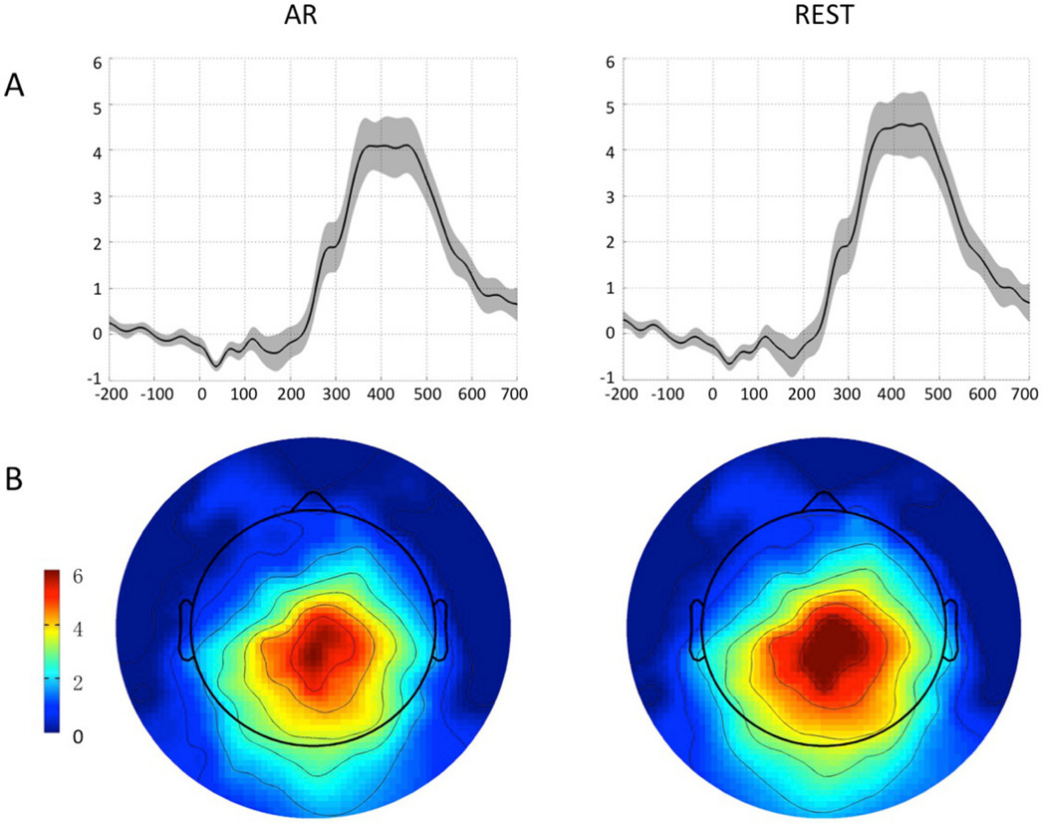
Comparison of ERP responses reconstructed by AR and REST. We assessed the reliability of the P300 response across sixteen subjects. (A) ERP signals and related standard error (in shaded grey) across subjects for the Pz electrode; (B) topography of the *Z-*values for the P300 peak intensity across subjects, estimated using a Wilcoxon Signed Ranks Test.

The quantitative analysis we conducted on ERP data from sixteen young healthy volunteers confirmed the results of our simulations. We calculated ERP responses at the Pz channel in each subject, as obtained either after AR or REST, and then performed a Wilcoxon Signed Ranks Test across subjects. The analysis of the time-courses revealed larger signal intensity for REST than AR, but also larger inter-subject variability (figure 7(A)). We then focused on the latency of maximum intensity, which ranged between 325 and 470 ms across subjects, and calculated channel-by-channel a Wilcoxon Signed Ranks Test on the corresponding signal intensity. We found a similar spatial distribution for AR and REST, with strongest activity over the parietal region. Nonetheless, *Z*-values were generally larger for REST compared to AR (figure 7(B)). Signal and noise levels for ERP data over the parietal region, as obtained after AR and REST, followed a similar trend (Spearman’s correlation, *r* = 0.815, 2-tailed *p* < 0.001 for signal; *r* = 0.876, 2-tailed *p* < 0.001 for noise). Signal amplitude using REST was significantly larger than the one using AR (Wilcoxon Signed Ranks Test, *Z* = 2.896, 2-tailed *p* = 0.004), whereas no significant difference between noise levels from REST and AR was found (*Z* = 1.758, 2-tailed *p* = 0.079) (see figure S7).

## Discussion

In this study we have assessed the most commonly used EEG re-referencing techniques, such as REST, AR and LMR, focusing in particular on the importance of using a high-density montage and a realistic head model. We primarily used simulated EEG data to examine the reconstruction accuracy in relation to electrode coverage, head model precision, uncertainty of electrode positions, and the presence of artifacts in the EEG data. We also analysed experimental high-density EEG data, thereby obtaining results largely consistent with those of our simulations. Notably, Yao previously conducted a comparative assessment of REST, AR and LMR using different EEG montages and head models (Yao 2001; Zhai and Yao 2004). In the paper introducing REST, 32-, 64- and 128-channel EEG systems were examined using a homogenous spherical head model; then, a comparison was made between a homogenous spherical head model and a three-layer spherical head model with 128-channel EEG only (Yao 2001). In a subsequent study, the performance of REST with a realistic head model was assessed in relation to AR and LMR. Specifically, the comparison was conducted using a BEM and a three-layer spherical head model, using a 128-channel EEG only (Zhai and Yao 2004). In both studies mentioned above, the effect of randomly distributed noise over the sensors was also evaluated. Accordingly, this manuscript expands on these previous studies in a number of ways: (1) in addition to the analysis of randomly distributed noise, we have investigated the effects of additional artifacts that are either localized or homogeneously distributed over the EEG sensors; (2) we have investigated the interaction between the number of EEG channels and the kind of head model used during referencing. This includes studying the reconstruction error of different re-referencing approaches for a 256-channel EEG system which is as yet not reported; (3) we have shown that electrode position inaccuracies can introduce additional errors for REST. This is an important consideration when evaluating its performance against that of AR and LMR, but it is also important that EEG practitioners who wish to use REST are aware that recording single subject electrode positions greatly improves the accuracy of the reconstruction and as such signal detection.

The general theory about the ideal EEG reference posits that it should be neutral, i.e. should contain no brain activity. Under this condition, true brain signals can be accurately recovered by re-referencing (Dien 1998). The comparison between the scalp map of simulated EEG data with those transformed in LMR, AR, and REST provided important insights into the performance of each re-referencing solution. In line with the previous literature (Dien 1998, Kayser *et al* 2007), our analysis revealed that CzR and LMR have substantially altered signal distributions (figures 3 and S3). The fact that LMR may induce a spatial shift of potentials towards frontal and central regions is particularly relevant to interpret a large number of studies that used this re-referencing solution for ERP studies (Light *et al* 2010, Horvath 2013, Zizlsperger *et al* 2014). Notably, it is not the first time that LMR is called into question, as it was previously reported that LMR can artificially inflate or deflate the correlation between electrodes (Dien 1998), and can induce the detection of primary visual responses over the mid-centroparietal region (Kayser *et al* 2007). Importantly, our analyses pointed out the fact that the EEG data re-referencing can lead to spatial distortions that depend not only on the dipole position, but also on its orientation. This result is largely consistent with the previous studies on the waveform distribution (Scherg 1990) and power sensitivity (Ahlfors *et al* 2010a) of EEG sources.

As for the performances of AR and REST, our qualitative analyses showed a very good correspondence with the simulated data. Nonetheless, more detailed, quantitative analyses clearly revealed that REST could provide more accurate reconstructions than AR (figure 3 and table 1) in a noise-free condition. The comparison between AR and REST across different regions of the scalp not only confirmed that the spatial distortions introduced by AR are generally bigger than REST, but also clarified that these distortions may either result from an over- or an under-estimation of the true potentials. Potential increases/decreases were largely varying, and depended both on the orientation of the dipole and its position with respect to the scalp (table 1). In this regard, it is worth noting that previous studies raised concerns about the use of AR when EEG sources were expected to be following the *z*- orientation, e.g. the auditory evoked N1 and P2 components (see for instance Tian and Yao 2013), because AR do not survey the bottom half of the head volume (Tomberg *et al* 1990, Dien 1998). Another important aspect to be considered is the consistency of the results from simulated data with those from experimental data. For instance, we revealed significantly stronger P300 signals over the parietal cortex using REST rather than AR. Along the same line, our simulations suggested that the use of AR leads to a more prominent potential attenuation than REST over this region (see table 2).

Since REST is calculated using the leadfield matrix (for more details, see equation (5)), its validity obviously depends on the accuracy of the head model. Extending previous studies that focused on REST either with a spherical (Yao 2001, Marzetti *et al* 2007) or a realistic (boundary element model, BEM) head model (Zhai and Yao 2004), we conducted a comparative analysis of the REST reconstruction error for a one-layer spherical, three-layer concentric spherical and a three-layer BEM constructed using the individual MR image of each subject. This last, more realistic head model provided the best performance on simulated data, especially for the high-density 256-channel EEG montage (figure 4), although it had relatively good performance also with a three-layer spherical model (Yao 2001, Zhai and Yao 2004). Our study is the first one that compared different re-referencing approach with a 256-channel EEG montage. It is worth noting that, this is rarely adopted in the current practice due to the relatively long preparation times and the volume conduction problem affecting neighbouring channels. Recent technological solutions are however overcoming part of these and other issues, so that high-density EEG might gain in the future a more widespread use in brain imaging research, as a potential alternative to magnetoencephalography (Michel and Murray 2012). As for the analysis of experimental data collected using a 256-channel EEG system, we observed a significantly larger ERP intensity, with no change in noise level, for REST with realistic head model compared to AR. Taken together, the results mentioned above highlight the importance of an accurate head model and a high-density montage when using REST.

It should be considered that not only an imprecise definition of the head model compartments, but also the uncertainty in the electrode locations can negatively impact on REST (Zhai and Yao 2004). Our results from simulated data are perfectly in line with this (see figures 4 and 5). To the best of knowledge, this is the first study to assess the impact of electrode misplacement on REST, although previous studies already showed that this reduces the accuracy of EEG source localisations (Van Hoey *et al* 2000, Wang and Gotman 2001). Importantly, our data showed that the impact of random and systematic electrode shifts is not negligible, and therefore needs to be considered when evaluating the performance of REST against that of AR and LMR. For instance, random and systematic position shifts above 2 mm introduced a cumulative error for REST larger than 5% (see figure 5). In turn, in the absence of electrode position shifts (figure 4), the difference in error between AR and REST with 256-channel EEG was below 5% regardless of the head model adopted. Based on this observation, we argue that AR may be as robust as REST for high-density EEG, particularly in the absence of precise electrode position information.

Many studies reported that the sampling and geometry of the EEG montage must be considered for the choice of the re-referencing approach (Yao 2001, Zhai and Yao 2004). These have a direct impact on the accuracy of AR and REST, but do not affect other approaches based on EEG channels at specific spatial locations, as for example LMR. AR has been previously recommended in the case of high-density EEG (Nunez *et al* 1997), based on the consideration that the average of all recorded EEG activity will be approximately null if the spatial sampling is both dense enough and sufficiently covers all the EEG signal space (Nunez and Srinivasan 2006). However, our analyses on AR across a large number of source positions suggested that a residual error of about 5% would still be present when using a 256-channel EEG system, and this error can go up to about 20% for a 10/20 standard EEG system (see figure 4). On the other hand, the RE obtained with REST (obtained in noise-free conditions and with a realistic head model) was typically lower then AR, and ranged between 0.6% (high-density EEG) and 14% (low-density EEG). Overall, our findings showed that having a high-density EEG montage is important for both AR and REST (figure 4) and suggested that REST with a realistic head model has superior reconstruction performance compared to AR. However, in the absence of reliable information about electrode positions and/or head tissue compartments, the reconstruction performance of REST may drop to a level comparable or even lower than AR for a 256-channel system (see figures 4 and 5). Notably, the use of a 256-channel system is most favourable for AR, which requires dense electrode sampling and wide coverage of the head. It should be mentioned that, in contrast to REST, AR is algorithmically simple, does not require the construction of a leadfield matrix and does not depend on its correctness. As such, we posit that there may be a clear advantage in the use of REST only in the specific situation of low channel account and availability of accurate information on head model and electrode positions.

It is worth noting that re-referencing approaches are not conceived to address the problem of artifacts inevitably present in the EEG data. However, we conducted a specific examination of the effect of temporal artifacts in our study, as we reasoned that this was an important element to guide the choice of the re-referencing technique in the analysis of real EEG data. Our results confirmed the idea that various re-referencing approaches may not be influenced by artifacts in the same manner. For instance, REST was found to be generally less sensitive than AR to different kinds of artifacts (figure 6). Additional errors in the reference estimation were substantially larger when the artifact was concentrated over a specific region of the scalp, as for example in the case of ocular artifacts. It is also important to consider that, on the other hand, a homogenously distributed artifact was completely cancelled by using AR, REST, and LMR. If the artifact has random distribution over the sensors, which is likely the case of hardware noise, AR and REST showed to be better solutions than LMR. Overall, our analyses suggested AR to be slightly more sensitive than REST to artifact-induced errors.

Although this study comprehensively investigated the importance of high-density EEG and realistic head model for REST and AR, there are still some limitations to be mentioned. First, we have built our simulations based on the Colin image, which is a high-resolution MR image often used in simulations studies (Collins *et al* 1998). This MR image of a human head is a template, and can be therefore considered as representative of many MR images of the same kind. However, it should be mentioned that we could have built our simulations based on any structural MR image, even one of our experimental subjects. Second, our study revealed the importance of using a realistic head model rather than spherical head models. Nonetheless, we investigated only a three-layer BEM head model for the group of realistic head models. Other realistic head models, such as finite element models (FEMs) or a finite difference models (FDMs), have been proposed (Hallez *et al* 2007). These are possibly more accurate than BEMs, in particular if the different conductivity of compact and spongy bone in the skull is considered (Akhtari *et al* 2002, Dannhauer *et al* 2011, Strobbe *et al* 2014). It is worth noting, however, that BEMs are currently implemented as default solution in the most used software tools for EEG analysis (e.g. SPM, FieldTrip, BrainStorm). Accordingly, we hope that our findings will be found useful by other researchers. Future developments in EEG volume conduction modelling are however warranted. Indeed, it should be considered that head models such as BEMs, FEMs and FDMs are at best approximate, and the nature of the error will be unknown until techniques to obtain in-vivo estimates of conductivity across the head become available (Seo and Woo 2014). Finally, we tested with our simulations the RE for AR and REST associated with four montages with different number of channels, as well as we studied the performances of these re-referencing techniques in the presence of three artifactual patterns. Future studies are warranted to assess the influence of EEG montages and artifactual patterns on the reconstruction performances of AR and REST.

## Conclusion

To the best of our knowledge, our study is the first to examine how much using a high-density montage and a realistic head model is essential for estimating a neutral reference on EEG signals. We observed that both AR and REST have relatively low reconstruction errors compared to LMR, and that REST is less sensitive than AR and LMR to artifacts mixed in the EEG data. For both AR and REST, high electrode density permits to achieve low re-referencing reconstruction errors. A realistic head model is critical for REST, leading to a more accurate estimate of a neutral reference compared to spherical head models. With a low-density (e.g. 21-channel or 71-channel) montage REST shows a more reliable reconstruction than AR either with a realistic or a three-layer spherical head model. Conversely, with a high-density (e.g. 256-channel) montage AR yields better results unless precise information on electrode positions is available. It is our hope that our quantitative investigation can help other brain imaging researchers in the choice of the most effective re-referencing approach for their future EEG studies.
